# Integrative single-cell RNA and ATAC sequencing reveals the impact of chronic cigarette smoking on lung epithelial responses to influenza and hyperoxia

**DOI:** 10.1186/s12931-025-03393-5

**Published:** 2025-12-09

**Authors:** Pei-Chun Cha, Zhenyang Zou, Jessica Nouws, Reginald M. Brewster, Charles S. Dela Cruz, Lokesh Sharma, Xiting Yan, Maor Sauler

**Affiliations:** 1 Department of Chronic Disease Epidemiology, Yale School of Public Health, New Haven, CT USA; 2 Department of Biostatistics, Yale School of Public Health, New Haven, CT USA; 3 Section of Pulmonary, Critical Care and Sleep medicine, Yale School of Medicine, 300 Cedar Street (S441 TAC), New Haven, CT 06520-8057 USA; 4 Division of pulmonary, allergy, critical care and sleep medicine, University of Pittsburgh, School of Medicine, Pittsburgh, PA USA

**Keywords:** ScRNA-seq, ScATAC-seq, Enrichment analysis, Lung epithelium, Smoking, Influenza, Hyperoxia

## Abstract

**Background:**

Cigarette smoke (CS) increases susceptibility to acute lung injury, yet how CS reshapes epithelial responses to subsequent insults remains unclear, and the extent to which aberrant epithelial responses are linked to epigenetic changes in vivo is uncertain.

**Methods:**

In this pilot, proof-of-principle study, we performed paired single-cell RNA-seq and ATAC-seq after chronic CS exposure followed by influenza or hyperoxia, generating joint expression–accessibility maps across epithelial subsets and inferring candidate transcriptional regulators.

**Results:**

Influenza and hyperoxia elicited distinct epithelial programs. Prior CS exposure amplified epigenetically regulated inflammatory signaling (cytokine/chemokine modules coupled to ERK/MAPK and PI3K) and suppressed epigenetically regulated reparative and differentiation pathways (γ-secretase/NOTCH). Transcription factor motif enrichment implicated ETS family factors and REL as candidate regulators of the CS-augmented responses in AT2 and ciliated cells.

**Conclusions:**

Paired single-cell RNA/ATAC profiling captures injury-conditioned epithelial programs and their epigenetic context after chronic CS exposure, demonstrating feasibility and providing a framework to prioritize targets of exposure-related aberrant responses.

**Supplementary Information:**

The online version contains supplementary material available at 10.1186/s12931-025-03393-5.

## Introduction

Acute respiratory distress syndrome (ARDS) is a life-threatening condition characterized by diffuse alveolar damage, increased vascular permeability, and severe hypoxemia. It can result from a variety of direct and indirect insults, including pneumonia, sepsis, aspiration, and trauma. While these triggers can initiate ARDS in otherwise healthy individuals, susceptibility and severity are strongly influenced by underlying factors such as advanced age, chronic lung disease, and other comorbidities. Because susceptibility varies sharply with these underlying conditions, defining the mechanisms that confer risk is essential to improving prevention and treatment [1].

Cigarette smoke (CS) exposure is a consistently observed risk factor for ARDS across diverse patient populations. Smokers who develop ARDS tend to be younger and have fewer comorbid conditions, which may reflect an underlying “primed” vulnerability [2–5]. In experimental models, CS exposure has been linked to an exaggerated inflammatory response and enhanced alveolar–capillary permeability [6, 7]. Beyond its direct cytotoxic effects, CS induces epigenetic modifications that may contribute to ARDS susceptibility [8–10]. For example, oxidative stress–driven activation of histone deacetylase 6 (HDAC6) disrupts cytoskeletal stability and increases vascular permeability [11]. However, the full spectrum of CS-induced epigenetic changes and their contribution to ARDS pathogenesis remain incompletely defined.

Advances in single-cell multi-omics now enable joint epigenetic and transcriptional profiling at single-cell resolution [12]. These approaches reveal not only gene expression patterns but also how chromatin accessibility governs transcriptional programs that direct cellular responses to injury. In this pilot study, we applied single-cell RNA sequencing (scRNA-seq) and ATAC sequencing (scATAC-seq) to assess how CS alters the epigenome and thereby shapes transcriptomic responses to acute injury. Using hyperoxia and influenza infection to model oxidative and immune-mediated injury, we investigated how prior CS exposure influences these responses. Through integrative multi-omic analysis, we tested the hypothesis that CS induces epigenetic alterations that can be detected and mechanistically linked to maladaptive responses to subsequent injury.

## Methods

### Animals

Male AKR/J mice (8–10 weeks old) were purchased from The Jackson Laboratory (Bar Harbor, ME, USA) and acclimated for one week prior to experimentation. Mice were housed under standard laboratory conditions with free access to food and water. They were assigned to six groups (*n* = 1 per group): control (CON), CS, hyperoxia (HYP), influenza infection (FLU), CS plus hyperoxia (CS + HYP), and CS plus influenza (CS + FLU). At the end of all exposure periods, mice were euthanized and lung tissues were collected as previously described [13]. Lungs were snap-frozen and stored in liquid nitrogen for further processing. All procedures were approved by the Yale University Institutional Animal Care and Use Committee (IACUC) and conducted in accordance with National Institutes of Health guidelines for the care and use of laboratory animals.

### CS exposure

Mice were randomized to receive mainstream smoke generated from 3R4F Kentucky reference cigarettes using the inExpose™ inhalation exposure system (SCIREQ, Montreal, Canada). Exposures were conducted in a whole-body chamber equipped with internal separators following the manufacturer’s standard configuration. Exposure protocols were run using the SCIREQ Flexiware software (7 puffs per cigarette, 35 mL puff volume, 2 s per puff). Per session, mice were exposed to 16 cigarettes, once daily, 5 days per week, for 32 weeks. After the final exposure, animals were rested for 72 h before receiving the subsequent injury.

### Influenza infection (PR8)

For influenza exposure, mice were lightly anesthetized with isoflurane and intranasally inoculated with 10 PFU of the H1N1 A/Puerto Rico/8/34 (PR8) strain in 50 µL sterile PBS, as previously described [14, 15]. Following inoculation, mice were returned to their home cages and monitored daily for signs of illness. Mice were euthanized 7 days post-infection.

### Hyperoxia

For hyperoxic injury, mice were placed in a sealed Plexiglas chamber and exposed to 100% oxygen for 48 h as previously described [16].

### Preparation of samples for sequencing libraries

Nuclei were isolated from snap-frozen lung tissue using the 10x Genomics Nuclei Isolation Kit, following the manufacturer’s protocol. Aliquots of the nuclei suspension were stained with 0.4% trypan blue and counted using a Thermo Fisher Countess II FL. Samples with > 90% viability were used for downstream analysis. Approximately 10,000 nuclei per sample were loaded onto chip J and processed with the Chromium X Series to create Gel Beads-in-Emulsion (GEMs). Subsequent steps were carried out according to the manufacturer’s instructions, including post-GEM-RT Dynabead cleanup and pre-amplification. Each sample was split into two portions: one for ATAC library construction and the other for cDNA amplification and gene expression library construction. DNA from the ATAC libraries (scATAC libraries) and cDNA from the gene expression libraries (scRNA libraries) were assessed at two time points using the Agilent Bioanalyzer High Sensitivity Chip to ensure quality. Six scATAC libraries and six scRNA libraries were sequenced on the NovaSeq 6000 platform using a 2 × 100 bp paired-end configuration. Data are available from the GEO database (GSE294221).

### Preprocessing of single-cell RNA sequencing data

Raw sequencing reads were preprocessed using the Cell Ranger pipeline (v7.1.0) with the mouse genome (mm10) for cell calling and read alignment to calculate the number of unique molecular identifiers (nUMI) for each gene. Downstream analyses were performed using Seurat (v5.1.0) [17]. Low-quality nuclei were excluded based on thresholds of > 200 detected features, >200 RNA counts, and < 5% mitochondrial gene content. Doublets were identified using DoubletFinder [18], and both doublets and debris were excluded through iterative clustering. The filtered dataset was log-normalized, scaled, and subjected to principal component analysis (PCA) for dimensionality reduction. Uniform Manifold Approximation and Projection (UMAP) was applied for visualization, and clustering was performed using the Louvain algorithm. Cell type annotations were assigned by integrating the dataset with a published reference [19] using FindTransferAnchors, followed by TransferData to map each cell to the most similar reference cell type, and then labeling clusters by the predominant cell type.

### Preprocessing of single-cell ATAC sequencing data

Raw sequencing reads were preprocessed using the Cell Ranger ATAC pipeline (v2.1.0) with mouse genome annotation (mm10) from 10x Genomics to identify peaks and calculate the number of UMIs per peak. Data were further processed using Seurat (v5.1.0) and the companion R package Signac (v1.13.1) [20]. Low-quality cells were removed from the aggregated scATAC-seq library (ATAC count > 400, nucleosome signal < 2, and TSS enrichment > 1). The filtered data were normalized using term-frequency inverse-document-frequency (TF-IDF).

### Identification of differentially expressed genes (DEGs) and differentially accessible peaks (DAPs)

To identify DEGs or DAPs between two given groups (CS vs. non-CS, influenza vs. non-influneza, hyperoxia vs. non-hyperoxia), we first filtered genes/peaks identified in at least 7.5% of cells in all samples. Since there is only one sample per group, we let $$\:{Y}_{ij}$$ denote the nUMI for gene/peak $$\:j$$ in cell $$\:i$$. Then we assumed that the nUMIs of gene/peak $$\:j$$ across all cells follows a negative binomial distribution: $$\:{Y}_{ij}\sim\:NB({S}_{i}{\mu\:}_{ij},\:{d}_{j})$$, where $$\:\mathrm{log}\left({\mu\:}_{ij}\right)={\beta\:}_{0j}+{\beta\:}_{1j}\cdot\:grou{p}_{i}$$, $$\:{S}_{i}$$ is the total number of UMIs in cell $$\:i$$, $$\:{d}_{j}$$ is the dispersion of the negative binomial distribution for gene/peak $$\:j$$. This model was fitted for each gene/peak separately to calculate the significance of $$\:{\beta\:}_{1j}$$. Genes with an FDR < 0.05 and an absolute log2 fold change (|$$\:{\beta\:}_{1j}$$|) > 0.5 were considered significant, while peaks were defined as significant based on a nominal *p*-value < 0.05 and an absolute log2 fold change (|$$\:{\beta\:}_{1j}$$|) > 0.5. To identify genes responding to the injury differently between smokers and non-smokers, i.e. the smoking-flu/hyperoxia interacted genes, we modified the model above by assuming $$\:\mathrm{log}\left({\mu\:}_{ij}\right)={\beta\:}_{0j}+{\beta\:}_{1j}\cdot\:smok{e}_{i}+{\beta\:}_{2j}\cdot\:fl{u}_{i}+{\beta\:}_{3j}\cdot$$$$\:smok{e}_{i}\cdot\:fl{u}_{i}$$. Significance of $$\:{\beta\:}_{3j}$$ was assessed and genes with an FDR < 0.05 and an absolute log2 fold change (|$$\:{\beta\:}_{3j}$$ |) > 0.5 were considered significant.

### Direct integration of scRNA-seq with scATAC-seq

To integrate scRNA-seq and scATAC-seq data, we focused exclusively on cells jointly profiled in both datasets, identified through shared cell barcodes. scATAC-seq peaks were assigned to genes using ChIPseeker (v1.38.0) [21, 22]. DEGs identified from scRNA-seq were paired with DAPs identified from scATAC-seq that were assigned to the same gene. A DEG–DAP pair was considered valid only when regulation was concordant: increased chromatin accessibility with upregulated expression, or decreased accessibility with downregulated expression.

### Transcription factor (TF) integration of scRNA-seq with scATAC-seq

We incorporated transcription factor binding site (TFBS) information to detect TFs relevant to DEG dysregulation. Promoter regions of DEGs were extracted using biomaRt (v2.58.2) [23, 24] (± 1 kb around the transcription start site), and TFBSs were identified using the JASPAR2020 database [25]. DEG–DAP pairs overlapping TFBSs in DEG promoter regions were retained. To prioritize key TFs, we calculated a TF enrichment score as follows: the number of binding sites of each TF was divided by the total number of TF binding sites across DEGs to calculate a baseline TF ratio (TFR_before). For DEG–DAP pairs (if > 25 per TF), the TF ratio was recalculated (TFR_after). The enrichment score was defined as TFR_after/TFR_before. For smoking–injury interactions, the sign of the enrichment score reflected the direction of regulation: positive if concordant, negative if discordant. The top 10 TFs were selected if their absolute enrichment score was ≥ 2; if fewer than 10 TFs met this threshold, only those that did were selected.

### Pathway enrichment analysis

DEGs from CS, influenza, and hyperoxia groups, as well as interaction genes, were subjected to pathway enrichment analysis using MetaCore. The enrichment tool was used to identify pathways involving these genes. Pathways were considered validated if enriched in validation datasets at nominal *p* < 0.05.

### Data validation

To validate pathways and TFs, we analyzed two datasets: GSE261627 (control *n* = 6, influenza infection *n* = 6, influenza infection + smoking *n* = 6) and GSE241468 (smoking *n* = 8, control *n* = 8) [26, 27]. For GSE261627, we used GEO2R https://www.ncbi.nlm.nih.gov/geo/info/geo2r.html to identify DEGs associated with influenza infection (A/Anhui/1/2013 (H7N9) virus) and their interactions with smoking. Influenza-infected samples were compared with controls to identify influenza-related DEGs, and influenza + smoking samples were compared with influenza alone to assess smoking effects. Genes further upregulated in influenza + smoking were classified as positive interactions, while those further downregulated were classified as negative interactions. DEGs were included if FDR < 0.05 and absolute log2 fold change > 0.5. TF enrichment was performed as above but based on DEGs alone. GSE241468 was analyzed using our established pipeline (DEG identification, DAP identification, DEG–DAP integration, and pathway analysis). For TF validation, enrichment was evaluated from DAPs alone (TFBS overlap within promoter-proximal DAPs). Pathways were considered validated if enriched in validation datasets at nominal *p* < 0.05.

## Results

### Cell annotation and joint visualization of scATAC-seq and scRNA-seq datasets

**Fig. 1:**
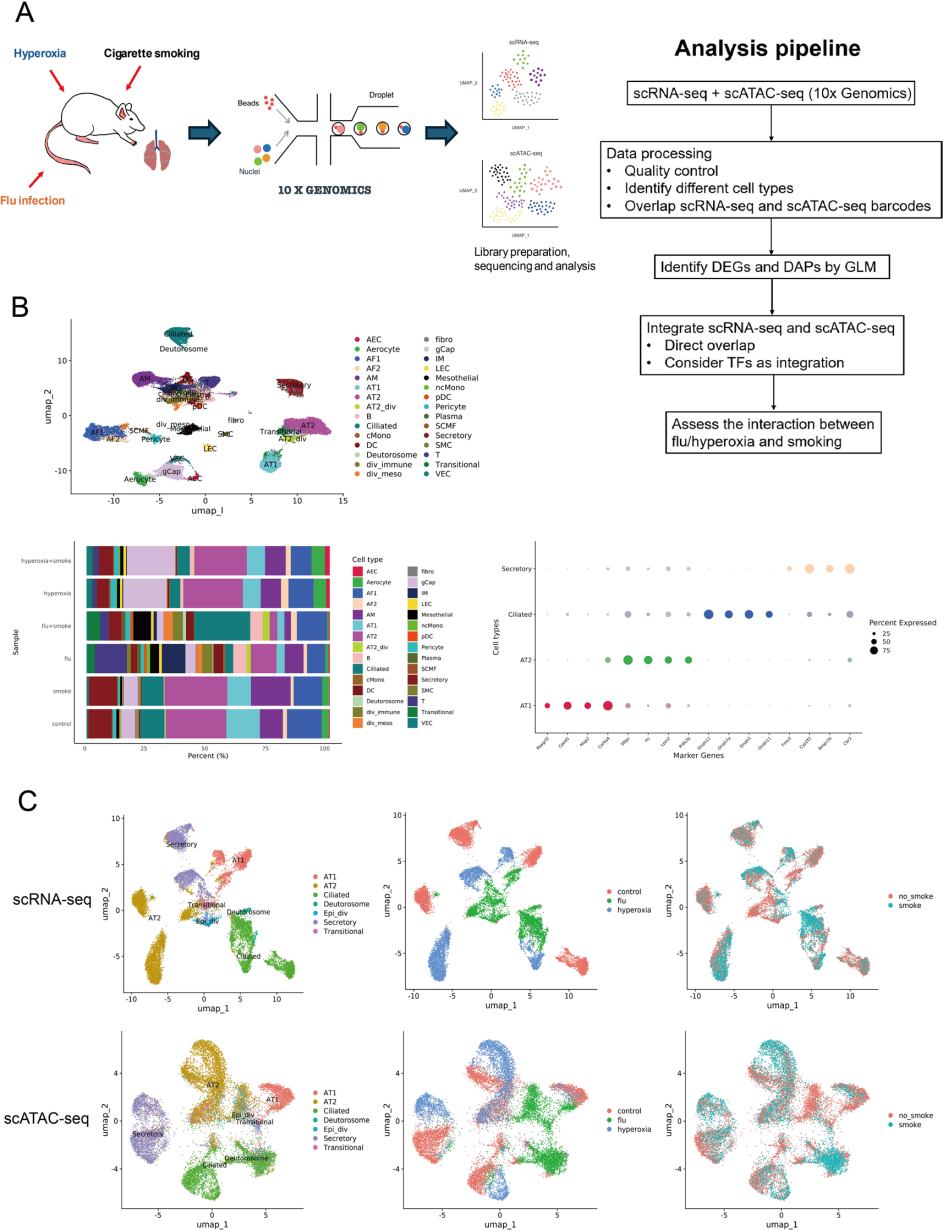
Workflow and cell-type annotation of integrated single-cell ATAC sequencing (scATAC-seq) and single-cell RNA sequencing (scRNA-seq) datasets. **A** Graphical abstract of experimental methodology and analysis pipeline. Mouse lung tissue was assessed with scRNA-seq and scATAC-seq (*n* = 1/group). Abbreviations: DEGs, differentially expressed genes; DAPs, differentially accessible peaks; GLM, generalized linear model; TFs, transcription factors. **B** Left top: Uniform Manifold Approximation and Projection (UMAP) visualization of single cells classified by scRNA-seq data. Left bottom: Proportions of cell types across each sample. Right bottom: Markers genes of epithelial cells. **C** UMAP plots of scRNA-seq and scATAC-seq dataset, clustered by cell types, condition (control, influenza (flu), hyperoxia), and smoking status

To examine the impact of CS exposure on susceptibility to acute lung injury, we generated matched scRNA-seq and scATAC-seq profiles from lung tissue of mice exposed to either CS or room air, followed by hyperoxia or acute influenza infection (Fig. 1A). This paired multiomic design enabled direct integration of transcriptional and chromatin-accessibility profiles from the same biological samples, providing a framework to define the molecular consequences of CS in the context of acute injury.

Across scRNA-seq libraries, we obtained a mean of 573,773,762 reads per lung (s.d. = 62,742,482) and 29,633 reads per cell (s.d. = 11,739) (Supplementary Table 1). After annotating cellular clusters (Fig. 1B, left), we focused on epithelial cells, given their relative abundance in our dataset and their role as the lung’s first line of defense against environmental insults. Within this compartment, we identified canonical subtypes, including alveolar type 1 (AT1), alveolar type 2 (AT2), ciliated, and secretory cells—based on expression of established marker genes (Fig. 1B, lower right). The composition of epithelial cell populations across experimental conditions is shown (Fig. 1B, Supplementary Table 2).

Across scATAC-seq libraries, we obtained a mean of 637,281,865 reads per library (s.d. = 118,797,857) and 40,732 reads per cell (s.d. = 6,760), with sequencing saturation and barcode metrics reported in Supplementary Table 3. Cell labels for scATAC-seq were transferred by mapping scATAC barcodes to matched, annotated scRNA-seq profiles from the same experimental condition. We retained only barcodes that satisfied quality criteria in both modalities. A joint UMAP of both scRNA-seq and scATAC-seq profiles demonstrated distinct clustering by cell type and condition (Fig. 1C).

### Hyperoxia and influenza induce coordinated transcriptional and chromatin-accessibility changes

**Fig. 2:**
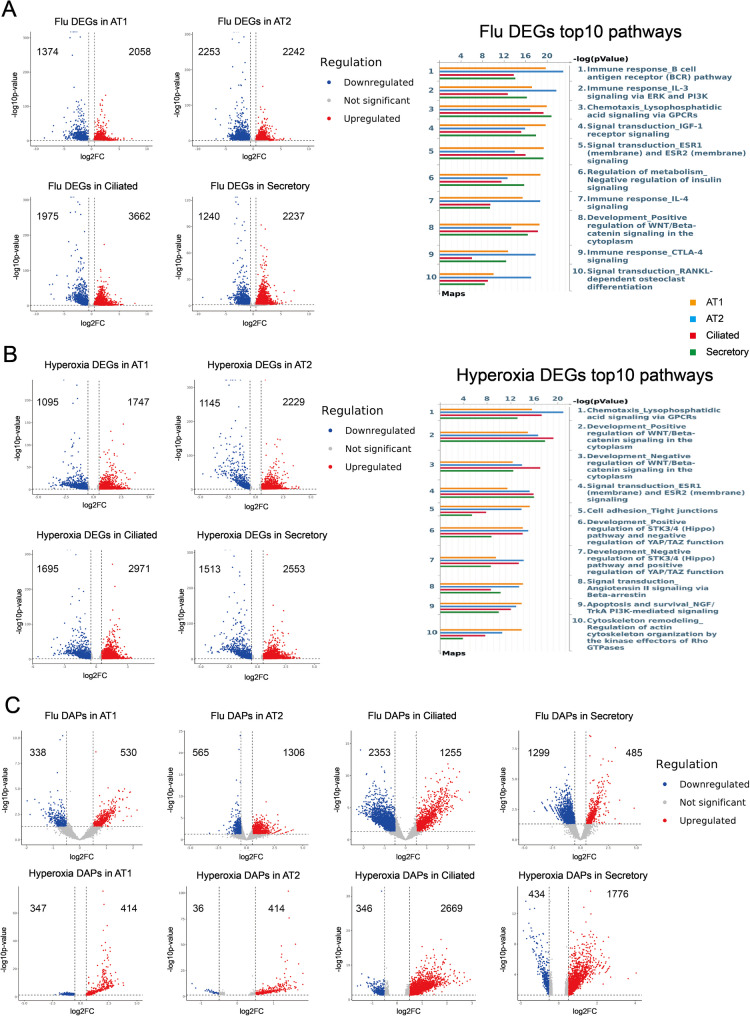
Differentially expressed genes (DEGs) from single-cell RNA sequencing (scRNA-seq) data and differentially accessible peaks (DAPs) from single-cell ATAC sequencing (scATAC-seq) in response to influenza infection and hyperoxia. **A** Left: Volcano plots displayed DEGs in response to influenza (flu) infection, showing upregulated and downregulated genes. Genes with an FDR < 0.05 and an absolute log2 fold change > 0.5 were considered significant. Genes with significant upregulation were highlighted with red, and those with significant downregulation were highlighted with blue. The numbers shown in the plots represented the number of genes that were upregulated or downregulated. Right: Top 10 enriched pathways from analysis of flu upregulated DEGs. Pathway enrichment analysis highlighted biological processes significantly associated with flu-affected genes. **B** Left: Volcano plots displayed the DEGs in response to hyperoxia, showing upregulated and downregulated genes. Genes with an FDR < 0.05 and an absolute log2 fold change > 0.5 were considered significant. Genes with significant upregulation were highlighted with red, and those with significant downregulation were highlighted with blue. The numbers shown in the plots represented the number of genes that were upregulated or downregulated. Right: Top 10 enriched pathways from analysis of hyperoxia upregulated DEGs. Pathway enrichment analysis highlighted biological processes significantly associated with hyperoxia-affected genes. **C** Top: Volcano plots displayed DAPs in response to flu infection, showing upregulated and downregulated peaks. Peaks were defined as significant based on a nominal *p*-value < 0.05 and an absolute log2 fold change > 0.5. Peaks with significant upregulation were highlighted with red, and those with significant downregulation were highlighted with blue. Bottom: Volcano plots displayed DAPs in response to hyperoxia, showing upregulated and downregulated peaks. Peaks were defined as significant based on a nominal *p*-value < 0.05 and an absolute log2 fold change > 0.5. Peaks with significant upregulation were highlighted with red, and those with significant downregulation were highlighted with blue

We first assessed transcriptional responses to influenza infection and hyperoxia (Fig. 2, Supplementary Data 1–4). Pathway enrichment analysis revealed distinct responses: influenza predominantly activated immune pathways, including antigen receptor pathway and cytokine signaling through ERK and PI3K pathways, whereas hyperoxia was enriched for chemotactic, cell adhesion, and developmental pathways such as LPA–GPCR chemotaxis, STK signaling, and WNT/β-catenin signaling.

**Fig. 3:**
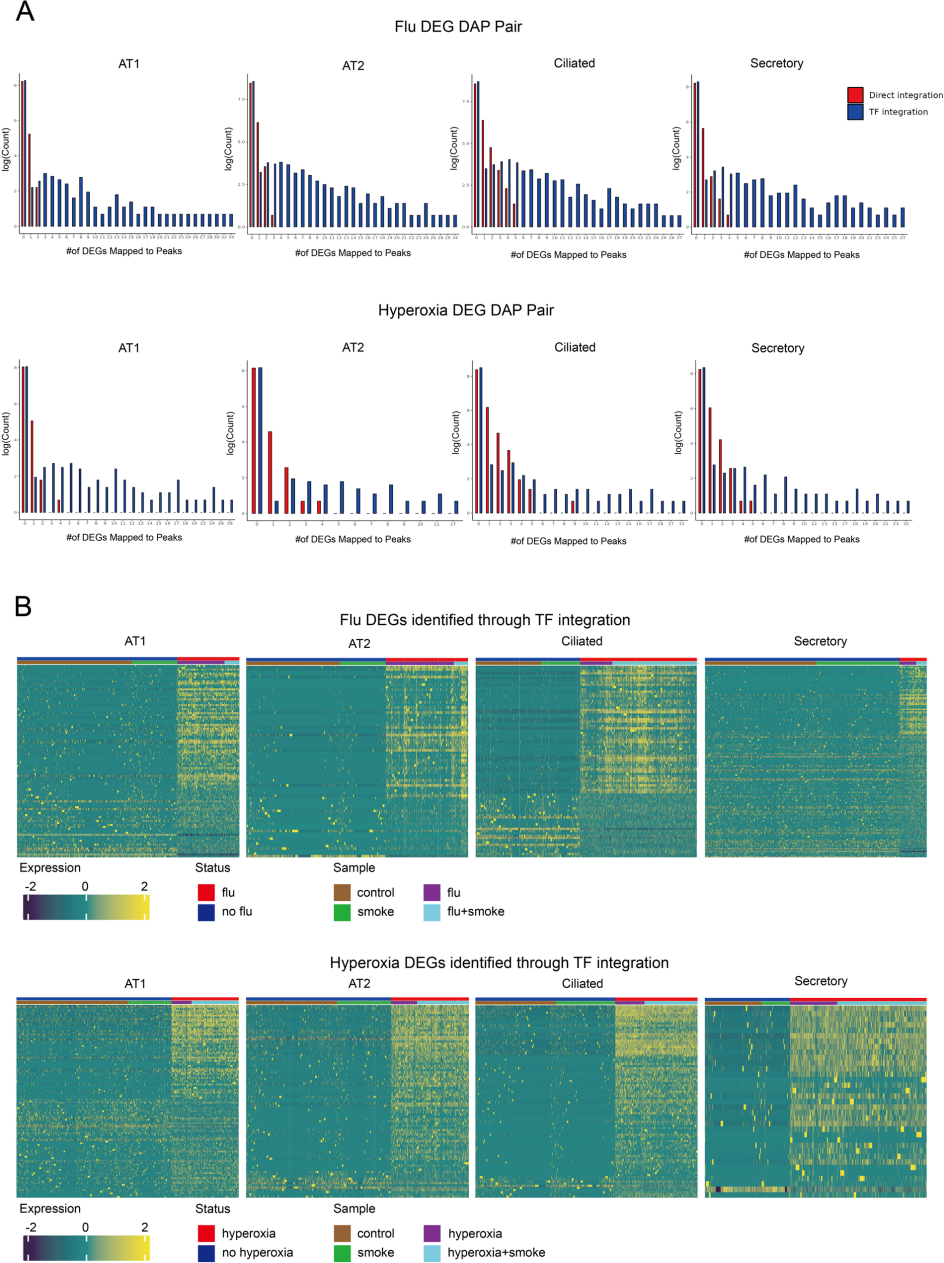
Integration of single-cell RNA sequencing (scRNA-seq) and single-cell ATAC sequencing (scATAC-seq). **A** Top: Histograms showing distribution of the number influenza (flu) differentially expressed genes (DEGs) linked per peak by direct and transcription factor (TF) integration. The X-axis represents the number of a specific DEG mapped to differentially accessible peaks (DAPs), while the Y-axis indicates the frequency of occurrences for each number. Bottom: Histograms showing distribution of the number hyperoxia DEGs linked per peak by direct and TF integration. The X-axis represents the number of a specific DEG mapped to DAPs, while the Y-axis indicates the frequency of occurrences for each number. **B** Top: Heatmaps displayed DEGs in response to influenza (flu) infection across four epithelial cell types, with the following numbers of upregulated and downregulated DEGs: AT1 (68/37), AT2 (217/59), ciliated (225/162), and secretory (63/91). Bottom: Heatmaps showed hyperoxia-responsive DEGs in the same four epithelial cell types, with the following numbers of upregulated and downregulated DEGs: AT1 (50/55), AT2 (30/3), ciliated (70/12), and secretory (76/9). Both analyses utilized TF integration to identify cell type-specific gene expression patterns associated with flu infection and hyperoxia exposure

To assess the relationship between changes in chromatin accessibility and gene expression, we then integrated the scATAC-seq and scRNA-seq datasets through direct and TF integration. Direct integration involved the identification of DEGs associated with nearby DAPs within a 1-kb window and retaining concordant DEG-DEP pairs (increased accessibility with upregulated expression; decreased accessibility with downregulated expression). For TF integration, DEG-DEP pairs were overlaid on known TFBS to identify potential regulatory elements mediating expression changes. Direct integration yielded the following counts of concordant DEG–DAP pairs **(**Fig. 3A, Supplementary Table 4): ciliated − 954 after influenza, 637 after hyperoxia; AT2–533 after influenza, 111 after hyperoxia; AT1–201 after influenza, 162 after hyperoxia; secretory – 329 after influenza, 505 after hyperoxia. Further integration of TF information reduced the number of DEG-DAP pairs by slightly less than 50% (Fig. 3A, Supplementary Table 4). The heatmaps of DEGs identified through TF integration were shown in Fig. 3B. Although this approach identified fewer DEG-DAP pairs than direct integration, it provided deeper insights into regulatory mechanisms.

### CS modifies epithelial transcriptional responses to influenza and hyperoxia

**Fig. 4:**
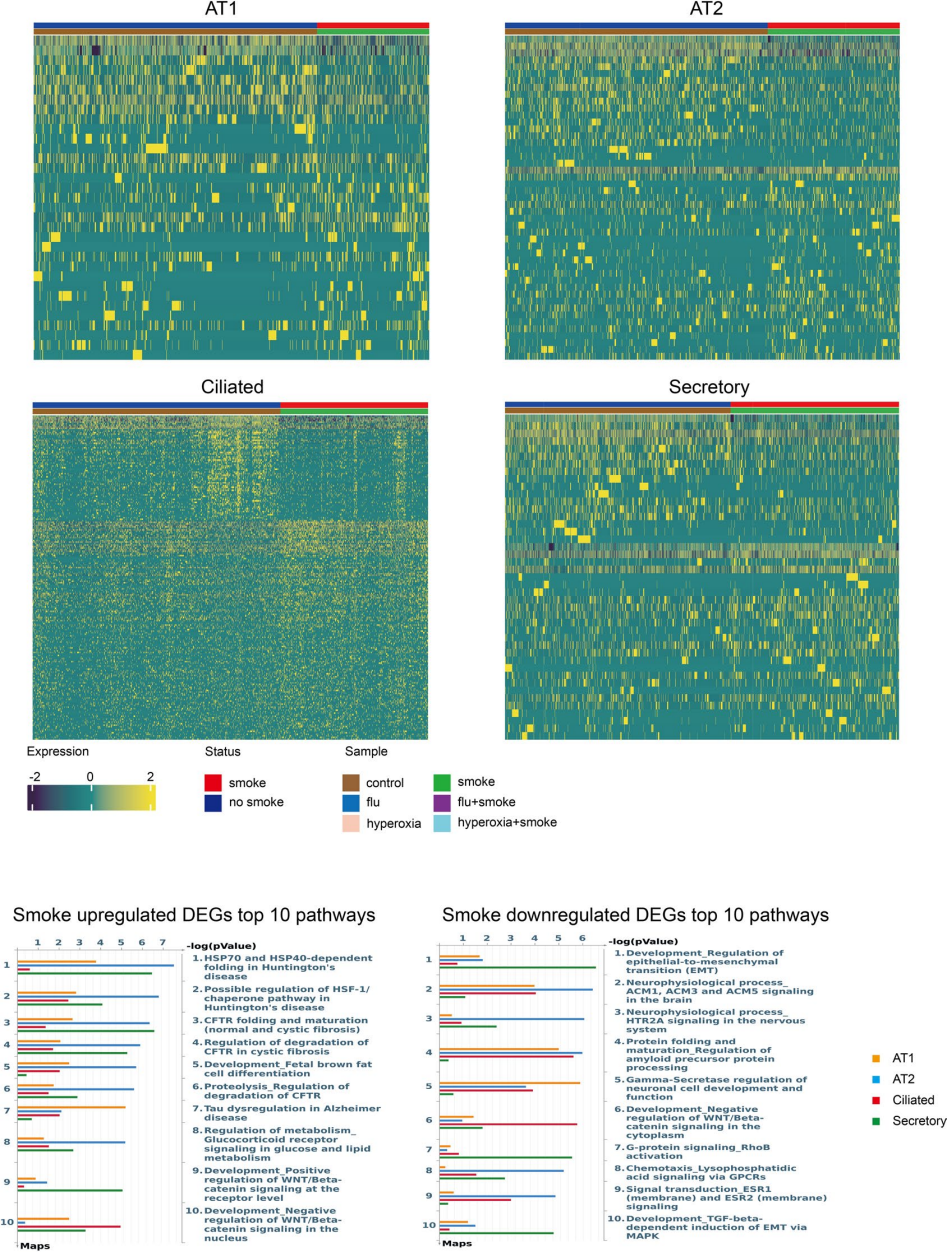
Differentially expressed genes (DEGs) from single-cell RNA sequencing (scRNA-seq) data in response to smoking. Top: Heatmap showing DEGs across four epithelial cell types in response to smoking, with the following numbers of upregulated and downregulated DEGs: AT1 (92/61), AT2 (69/49), ciliated (225/153), and secretory (67/61). Genes with an FDR < 0.05 and an absolute log2 fold change > 0.5 were considered significant Bottom: Pathway enrichment analysis of smoking-associated DEGs. Top 10 pathways enriched for positively regulated DEGs are displayed on the left, while top 10 pathways enriched for negatively regulated DEGs are displayed on the right. *P*-values indicate the significance of pathway enrichment

We then sought to determine the impact of smoking on epithelial responses to injury. First, we assessed epithelial transcriptional changes following CS exposure alone. As expected, the transcriptional impact of CS was modest compared with either hyperoxia or influenza infection. Pathway analysis of DEGs revealed induction of heat-shock and proteostasis programs, consistent with ER/secretory-stress adaptation. In parallel, epithelial repair and remodeling programs were suppressed, including downregulation of EMT, RhoB-dependent cytoskeletal remodeling, LPA-driven chemotaxis, and the γ-secretase pathway that governs NOTCH signaling (Fig. 4). Parasympathetic (muscarinic) and serotonergic pathways were also attenuated, and WNT signaling was bidirectionally dysregulated, with components represented among both enriched and attenuated pathways. Notably, the number of DEG–DEP pairs was very low (*n* $\:\le\:$ 25), too few to meaningfully assess the impact of chromatin remodeling on gene expression following CS exposure alone (Supplementary Table 5, Supplementary Data 5–6).

**Fig. 5:**
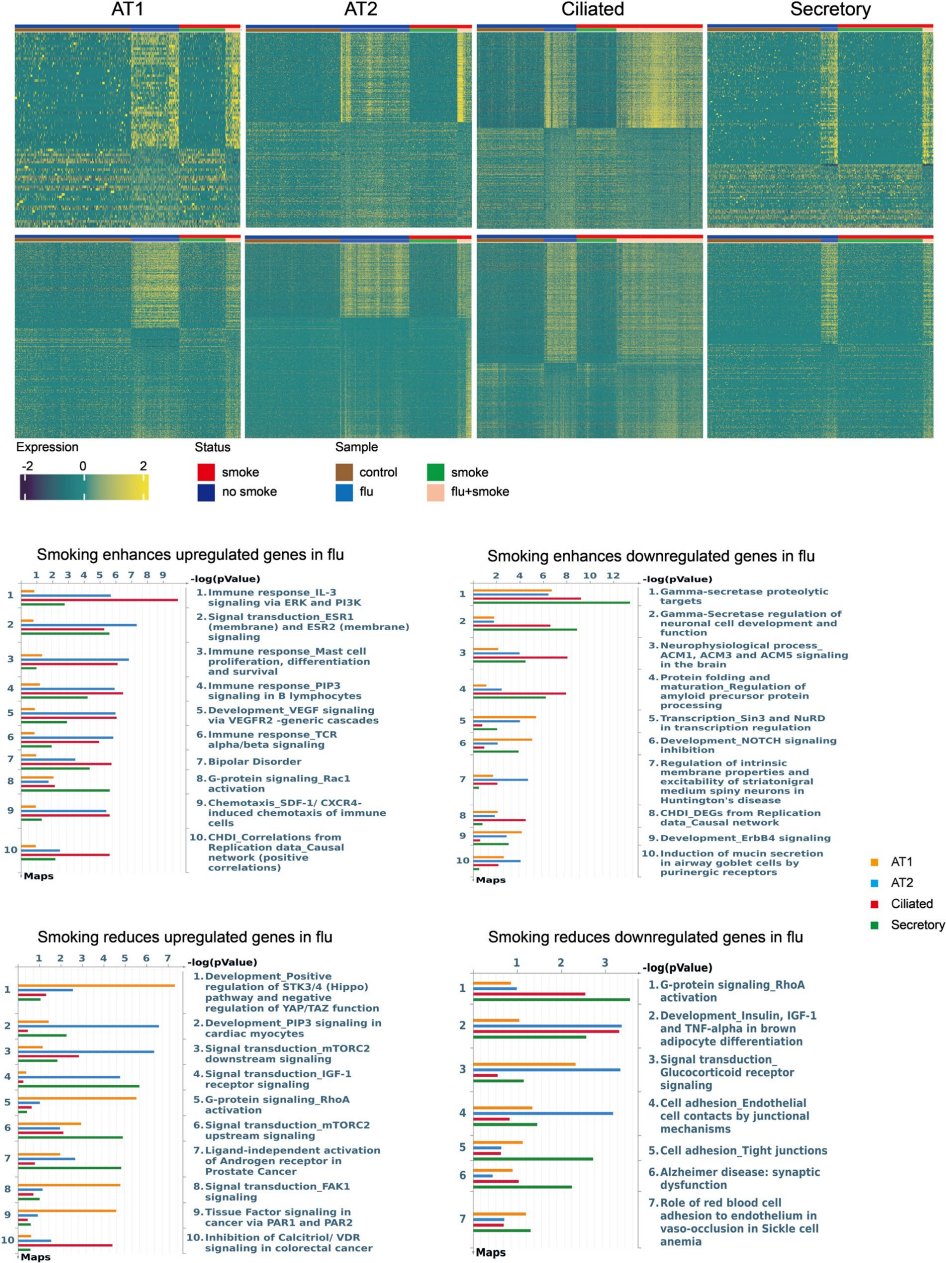
Pathway enrichment for smoking–influenza interactions differentially expressed genes (DEGs). Top: Heatmaps display genes with a significant interaction between smoking and influenza (flu) across AT1, AT2, ciliated, and secretory cells. Positive interaction = smoking enhances flu-up genes or further suppresses flu-down genes; negative interaction = smoking attenuates flu-up genes or mitigates flu-down genes. Interaction significance was tested in a generalized linear model (FDR < 0.05; |log2FC| >0.5); only genes that were DEGs in the flu contrast were retained as interacting. Counts per cell type: enhance flu-up—AT1 (46), AT2 (126), ciliated (226), secretory (89); enhance flu-down—AT1 (235), AT2 (489), ciliated (297), secretory (137); reduce flu-up—AT1 (188), AT2 (306), ciliated (461), secretory (166); reduce flu-down—AT1 (36), AT2 (175), ciliated (247), secretory (42) Bottom: Pathway enrichment for positive and negative interaction gene sets in each epithelial subtype. *P*-values indicate the significance of pathway enrichment

We then sought to determine how CS alters epithelial responses to influenza and hyperoxia. Pathway analysis of epithelial transcriptional responses demonstrated CS amplified inflammatory signaling while suppressing repair programs during influenza infection **(**Fig. 5). Epithelial cells from smoke-exposed lungs showed greater upregulation of cytokine and chemokine signaling coupled to downstream ERK/PI3K signaling cascades. These gene sets included IL-3 signaling, SDF-1/CXCR4-chemotaxis, mast cell activation, T-cell/B-cell receptor signaling suggestive of a broad upregulation of inflammatory pathways. Conversely, key regenerative and differentiation programs were blunted by CS exposure; CS deepened repression of Notch signaling (γ-secretase/Notch regulation and Notch-inhibition) and blunted flu-induced upregulation of key repair pathways, including Hippo-YAP/TAZ, mTORC2 and PI3K–AKT signaling. Additionally, neuron-associated signaling modules (muscarinic cholinergic signaling, synaptic excitability) were further suppressed as was mucin secretion by purinergic receptors, implying blunted ATP-driven mucus release and loss of mucociliary clearance. Other dysregulated pathways included adhesion and motility modules (FAK1, RhoA, tight-junctions) and hormone-receptor signaling pathways (insulin/IGF-1, Erbb4, Tissue Factor signaling, glucocorticoid-receptor signaling).

**Fig. 6:**
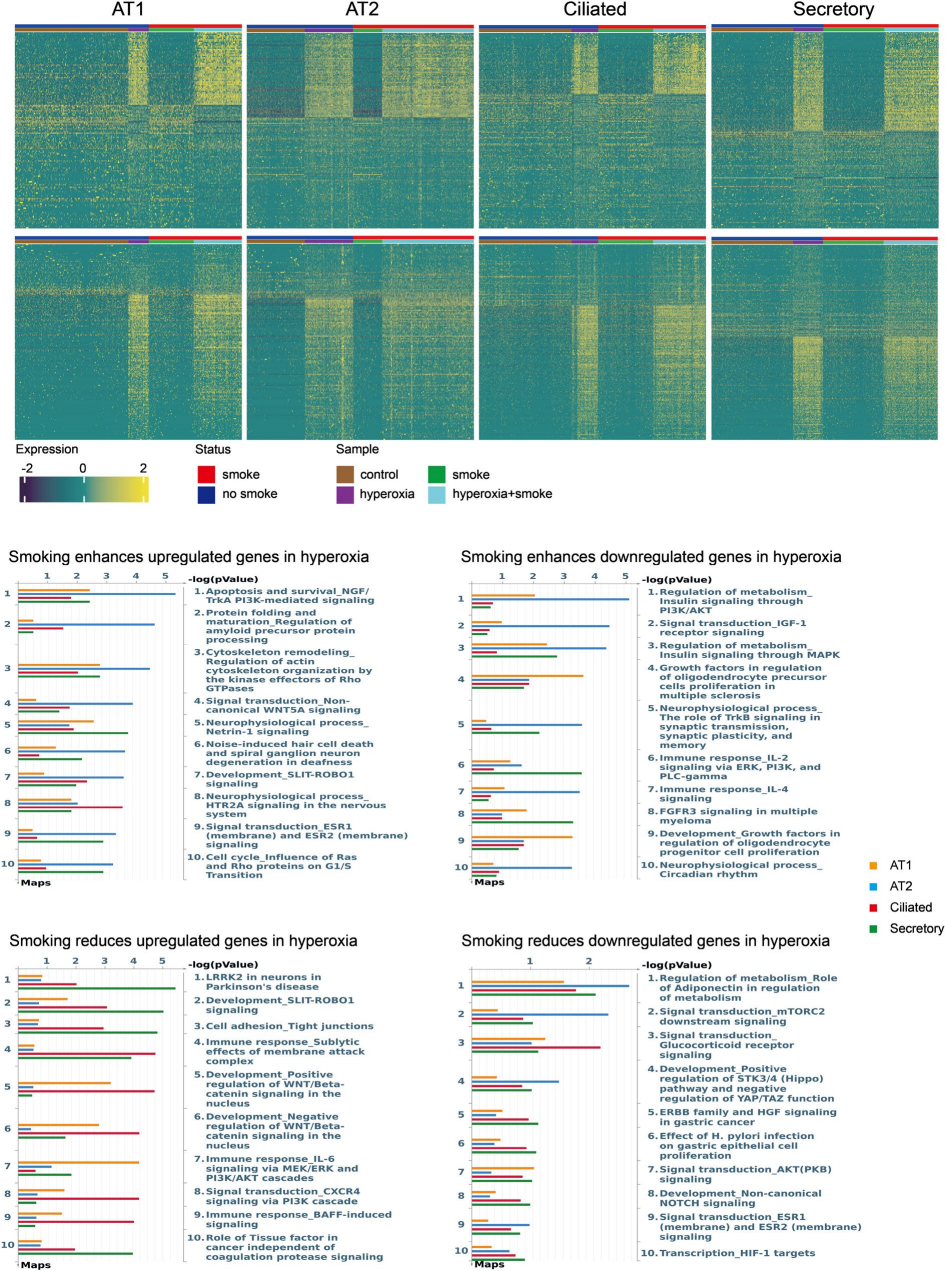
Pathway enrichment for smoking–hyperoxia interaction differentially expressed genes (DEGs). Top: Heatmaps display genes with a significant interaction between smoking and hyperoxia across AT1, AT2, ciliated, and secretory cells. Positive interaction = smoking enhances hyperoxia-up genes or further suppresses hyperoxia-down genes; negative interaction = smoking attenuates hyperoxia-up genes or mitigates hyperoxia-down genes. Interaction significance was tested in a generalized linear model with a smoke×hyperoxia term (FDR < 0.05; |log2FC| >0.5); only genes that were DEGs in the hyperoxia contrast were retained as interacting. Counts per cell type: enhance hyperoxia-up - AT1 (89), AT2 (100), ciliated (127), secretory (90); enhance hyperoxia-down 0- AT1 (52), AT2 (76), ciliated (57), secretory (91); reduce hyperoxia-up - AT1 (148), AT2 (116), ciliated (212), secretory (148); reduce hyperoxia-down - AT1 (51), AT2 (44), ciliated (97), secretory (131) Bottom: Pathway enrichment for positive and negative interaction gene sets in each epithelial subtype. *P*-values indicate the significance of pathway enrichment

Previous exposure to CS also dysregulated multiple pathways during hyperoxia including (Fig. 6): (1) cell-survival/stress signaling such as NGF/TrkA, PI3K–AKT, and mTORC2–AKT, (2) developmental/differentiation programs including canonical WNT/β-catenin and Notch signaling; (3) metabolic and endocrine pathways such as insulin/IGF and adiponectin signaling; and (4) immune and cytokine signaling. Collectively, these data suggested smoking enhanced survival signaling while broadly altering developmental, metabolic, and inflammatory signaling pathways after hyperoxia.

### CS alters chromatin accessibility to modulate epithelial responses to influenza

**Fig. 7:**
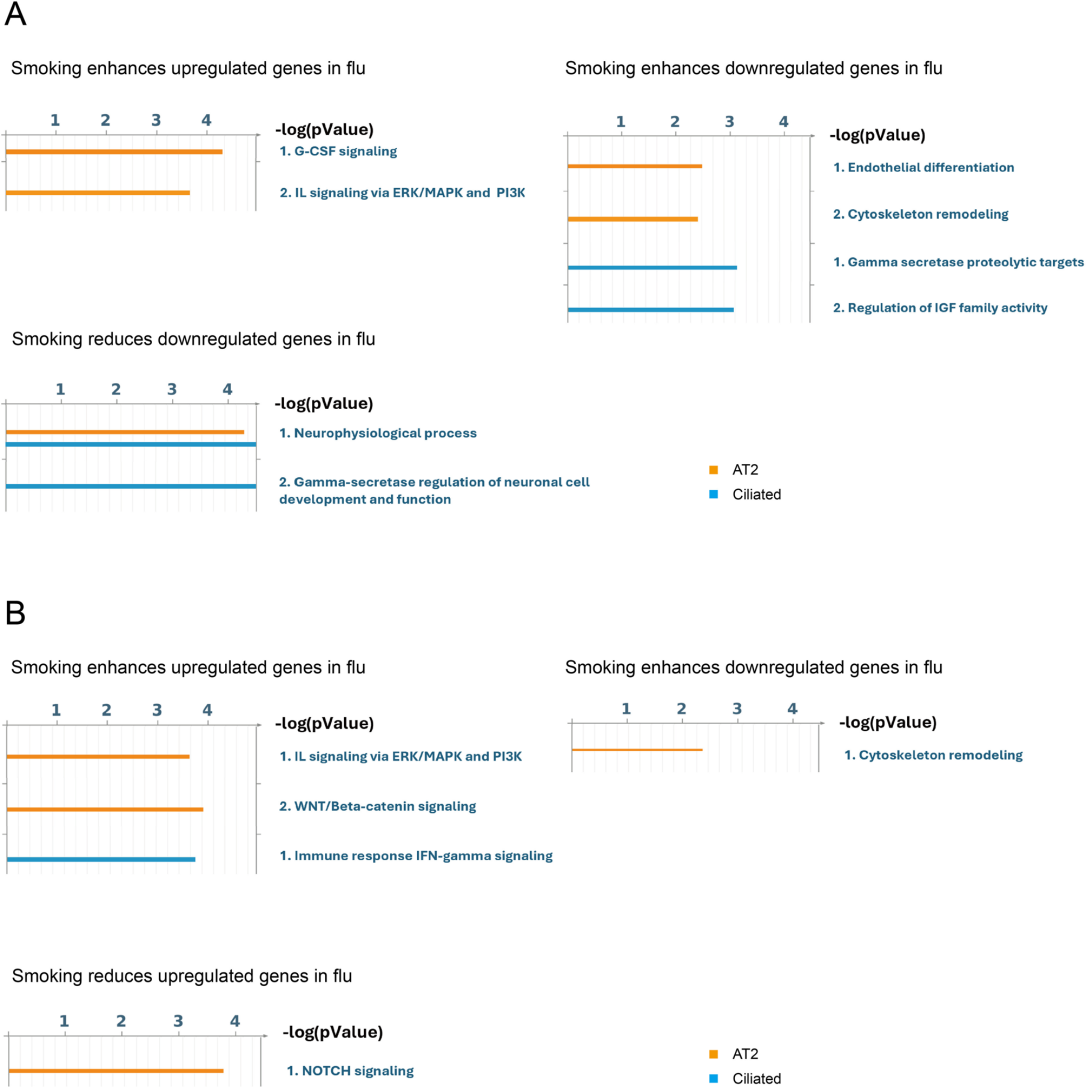
Pathway enrichment based on concordant DEG–DAP pairs. **A** Pathways enriched among smoking–influenza interaction genes defined by concordant DEG–DAP pairs in our study that overlap with GSE261627. **B** Pathways enriched among smoking–influenza interaction genes defined by concordant DEG–DAP pairs in our study that overlap with GSE241468 (DEG-DAP pairs). Conditions are denoted as: up reduced (CS exposure suppresses flu-upregulated genes), up enhanced (CS exposure further increases flu-upregulated genes), down reduced (CS exposure restores or partially restores expression of flu-downregulated genes), and down enhanced (CS exposure further decreases flu-downregulated genes). *P*-values indicate the significance of pathway enrichment

We then sought to assess how CS exposure modulates chromatin accessibility and thereby shapes epithelial transcriptional responses after influenza infection. Interaction testing was restricted to AT2 and ciliated cells following influenza infection, where > 25 gene–peak pairs were detected, while AT1 and secretory populations following influenza infection and all epithelial cells exposed to hyperoxia were excluded due to insufficient coverage (Supplemental Tables 6–7, Supplementary Data 7–10) We next performed pathway analysis on genes paired with a DAP to identify transcriptional changes directly linked to epigenetic remodeling. To improve the robustness of our findings, we further filtered results to include only those also dysregulated in an independent dataset of microarrays comparing air- and CS-exposed mice followed by A/H7N9 infection (GSE261627) or in AT2 cells from smokers (GSE241468) (Supplementary Data 11). Pathways associated with changes in chromatin accessibility included amplified cytokine and inflammatory signaling, specifically increased G-CSF, IL signaling cascades, and downstream ERK/MAPK, PI3K, and RAC1-dependent G-protein modules. In contrast, key repair and barrier pathways were suppressed, including endothelial differentiation, cytoskeletal remodeling, and the γ-secretase/NOTCH axis. IGF-family signaling was also reduced, consistent with impaired epithelial survival and regenerative capacity (Fig. 7, Supplementary Fig. 1–2).

### Potential TFs related to smoking influenza interaction

**Fig. 8:**
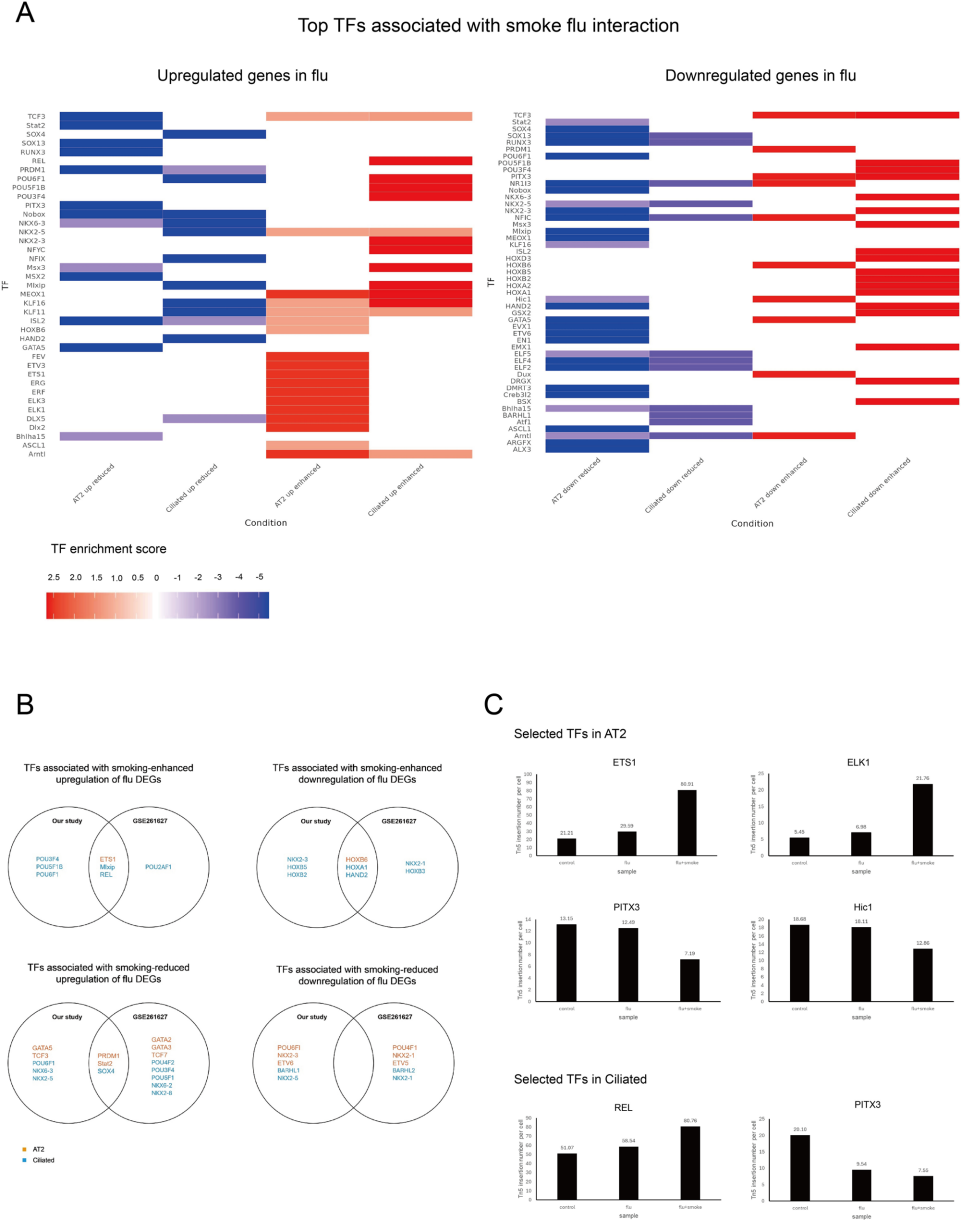
Transcription factor (TF) integration reveals regulatory mechanisms of smoking–influenza interactions. **A** Top TFs for each cell type, selected based on the highest TF enrichment score, with a minimum score threshold of 2. Conditions are denoted as: up reduced (CS exposure suppresses flu-upregulated genes), up enhanced (CS exposure further increases flu-upregulated genes), down reduced (CS exposure restores or partially restores expression of flu-downregulated genes), and down enhanced (CS exposure further decreases flu-downregulated genes). **B** Common TFs identified when compared to GSE261627 **C** Tn5 insertion number normalized by cell number of selected TFs that were also identified as amongst DAPs in GSE241468

We similary sought to identify changes in TF accessibility modified by CS exposure (Fig. 8A). TF integration revealed that prior CS exposure enhanced activity of ETS family (ETS1, ELK1, ELK3) in AT2 cells following influenza and Mlxip and REL in ciliated cells, collectively suggesting reinforcement of immune response. Prior CS exposure reduced activity of key epithelial regulators including SOX4 and further suppressed the activity of PITX3, HIC1, and HOX family TFs. We sought to validate these findings using both validation datasets (GSE261627, GSE241468). Overlap with TF enrichment performed on DEGs from GSE261627 redemonstrated similar changes in ETS1, REL, and SOX4 (Fig. 8B). Overlap with TF enrichment performed on DAPs from GSE24148 demonstrated similar overlap in ETS1, ELK1, REL, HIC1, and PITX3 (Fig. 8C, Supplementary Tables 8–9).

## Discussion

In this pilot study, we characterized gene expression and chromatin accessibility in epithelial cells to explore how chronic CS exposure shapes responses to acute injury. Through integrative single-cell RNA and ATAC sequencing, we uncovered a landscape in which CS amplifies inflammatory programs while blunting epithelial repair and differentiation in response to influenza infection and hyperoxia. These dual effects were mediated, at least in part, by altered chromatin accessibility and TF activity.

Prior studies in mice have shown that CS increases susceptibility to acute lung injury (ALI) [28–30], typically using “double-hit” models of CS exposure followed by lipopolysaccharide challenge. However, the mechanisms remain unclear. Here, we applied a similar approach in AKR mice, a strain particularly susceptible to CS. While we did not assess physiologic outcomes, we identified a transcriptional signature of CS marked by augmented pro-inflammatory pathways and impaired epithelial repair and differentiation. Only a subset of these changes coincided with altered chromatin accessibility. In AT2 and ciliated cells, prior CS induced chromatin remodeling that enhanced influenza-induced cytokine modules, including G-CSF and interleukin programs via ERK/MAPK, PI3K, and RAC1, with TF integration implicating ETS family members (ETS1/ELK1) and REL as candidate regulators [31–33]. Conversely, CS was associated with epigenetic silencing of NOTCH signaling and cytoskeletal remodeling, consistent with deficits in epithelial differentiation. Thus, CS-driven chromatin remodeling may establish a pro-inflammatory regulatory state that is unmasked by acute injury, a mechanism relevant to the morbidity and decline driven by exacerbations in chronic lung disease.

This is, to our knowledge, the first proof-of-principle study applying paired single-cell RNA/ATAC sequencing to examine how CS reprograms injury responses. The major limitation is the small sample size, compounded by use of both sexes; with only one mouse per exposure group, inter-animal variability could not be assessed, and findings should be viewed as hypothesis-generating rather than definitive. In addition, we analyzed a single post-injury time point, limiting our ability to track dynamic transcriptional and epigenetic changes or to distinguish early injury from late reparative responses. More broadly, multiomic integration remains an evolving field with technical and analytical constraints. Although we leveraged direct and TF-based approaches to connect chromatin accessibility with gene expression, these rely on assumptions about regulatory proximity and TF-binding annotation that may not fully capture chromatin architecture or cell-type–specific regulation. Nonetheless, we observed similar patterns in external cohorts, including increased accessibility at inflammatory genes and reduced accessibility at reparative pathways in human AT2 cells from smokers. These accessibility changes may underlie chronic inflammation and impaired tissue repair in COPD. Importantly, because chronic CS alone produces relatively modest transcriptional shifts, multiomic interrogation under superimposed injury provides a framework for identifying disease-relevant mechanisms that drive clinically meaningful exacerbations.

## Conclusion

In conclusion, our findings highlight how CS reshapes the transcriptional and regulatory landscape of epithelial cells during acute lung injury. Larger cohorts, temporal resolution, and further refinement of integrative models will be essential to fully realize the potential of multiomic approaches in dissecting complex lung disease mechanisms.

## Supplementary Information

Supplementary Material 1.

Supplementary Material 2.

Supplementary Material 3.

## Data Availability

The murine scRNA-seq and scATAC-seq data generated in this study are available from the GEO database (GSE294221), as well as the validation datasets (GSE261627 and GSE241468). All other data in the manuscript is available through the responsible corresponding author.

## Abbreviations

ALI: Acute lung injury
ARDS: Acute respiratory distress syndrome
CON: Control
COPD: Chronic obstructive pulmonary disease
CS: Cigarette smoke
DAP(s): Differentially accessible peak(s)
DEG(s): Differentially expressed gene(s)
FLU: Influnenza
HYP: Hyperoxia
LPA: Lysophosphatidic acid
scRNA-seq: Single-cell RNA sequencing
scATAC-seq: Single-cell ATAC sequencing
STK: Src family tyrosine kinases
TF(s): Transcription factor(s)
UMAP: Uniform Manifold Approximation and Projection
