# Supplementary material for: Integrative single-cell RNA and ATAC sequencing reveals the impact of chronic cigarette smoking on lung epithelial responses to influenza and hyperoxia
Source: Respir Res. 2025 Dec 9;27:13. doi: 10.1186/s12931-025-03393-5 (PMC12801856; doi:10.1186/s12931-025-03393-5)
Supplement: Supplementary file 1 — Supplementary Material 1. [file 12931_2025_3393_MOESM1_ESM.docx]

**Supplementary Information**

**Integrative Single-Cell RNA and ATAC Sequencing Reveals the Impact of Chronic Cigarette Smoking on Lung Epithelial Responses to Influenza and Hyperoxia**

Pei-Chun Cha^1†^, Zhenyang Zou^2†^, Jessica Nouws^3^, Reginald M. Brewster^3^, Charles S Dela Cruz^3,4^, Lokesh Sharma^3,4^, Xiting Yan^2,3^, Maor Sauler^3*^

^1^ Department of Chronic Disease Epidemiology, Yale School of Public Health, New Haven, Connecticut, USA

^2^ Department of Biostatistics, Yale School of Public Health, New Haven, Connecticut, USA

^3^ Section of Pulmonary, Critical Care and Sleep medicine, Yale School of Medicine, New Haven, Connecticut, USA

^4^ Division of pulmonary, allergy, critical care and sleep medicine, University of Pittsburgh, School of Medicine, Pittsburgh, PA, USA

†These authors are listed as co-first authors.

*** Corresponding author**

Maor Sauler,

Section of Pulmonary, Critical Care and Sleep medicine

Yale University School of Medicine,

300 Cedar Street (S441 TAC),

New Haven, CT 06520-8057, USA.

[maor.sauler@yale.edu](mailto:maor.sauler@yale.edu)

**Keywords**: scRNA-seq, scATAC-seq, enrichment analysis, lung epithelium, smoking, influenza, hyperoxia.


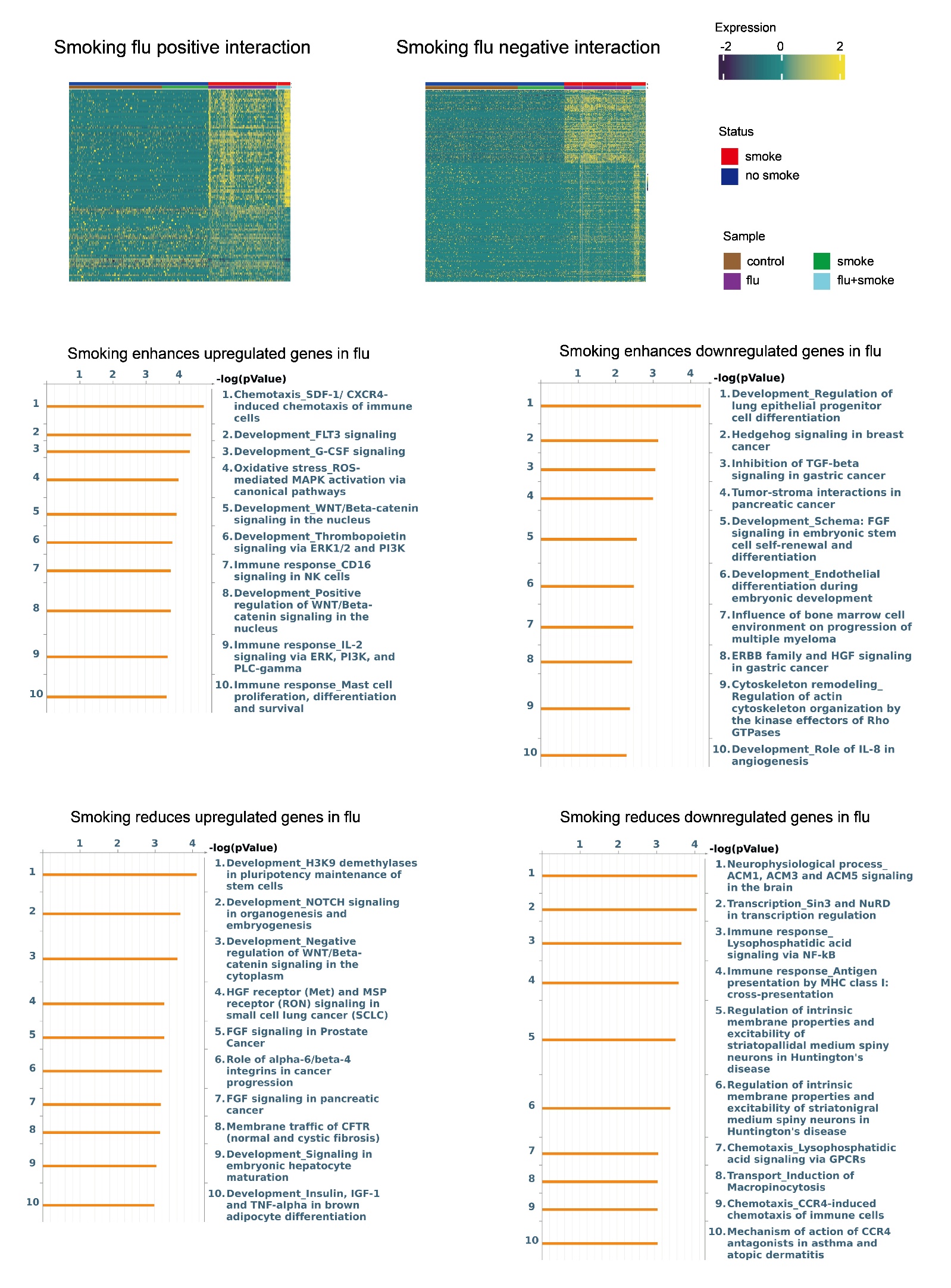


**Supplementary Fig. 1. Direct scRNA–scATAC integration identifies smoking–influenza interactions in AT2 cells.**Top: Heatmaps show smoking–influenza (flu) interaction genes in AT2 cells. Positive interactions (left panels) include cases where smoking enhances the expression of flu-upregulated genes or further suppresses flu-downregulated genes. Negative interactions (right panels) include cases where smoking dampens flu-upregulated genes or counteracts flu-downregulated genes. Significant interaction genes were defined as those differentially expressed in response to flu and further modified by smoking (FDR < 0.05, |log2 fold change| > 0.5). Numbers of interaction genes: smoking enhances flu-up genes (78), enhances flu-down genes (57), reduces flu-up genes (87), reduces flu-down genes (145).

Bottom: Pathway enrichment analysis of positively and negatively interacting smoke–influenza genes. P-values indicate the significance of pathway enrichment.


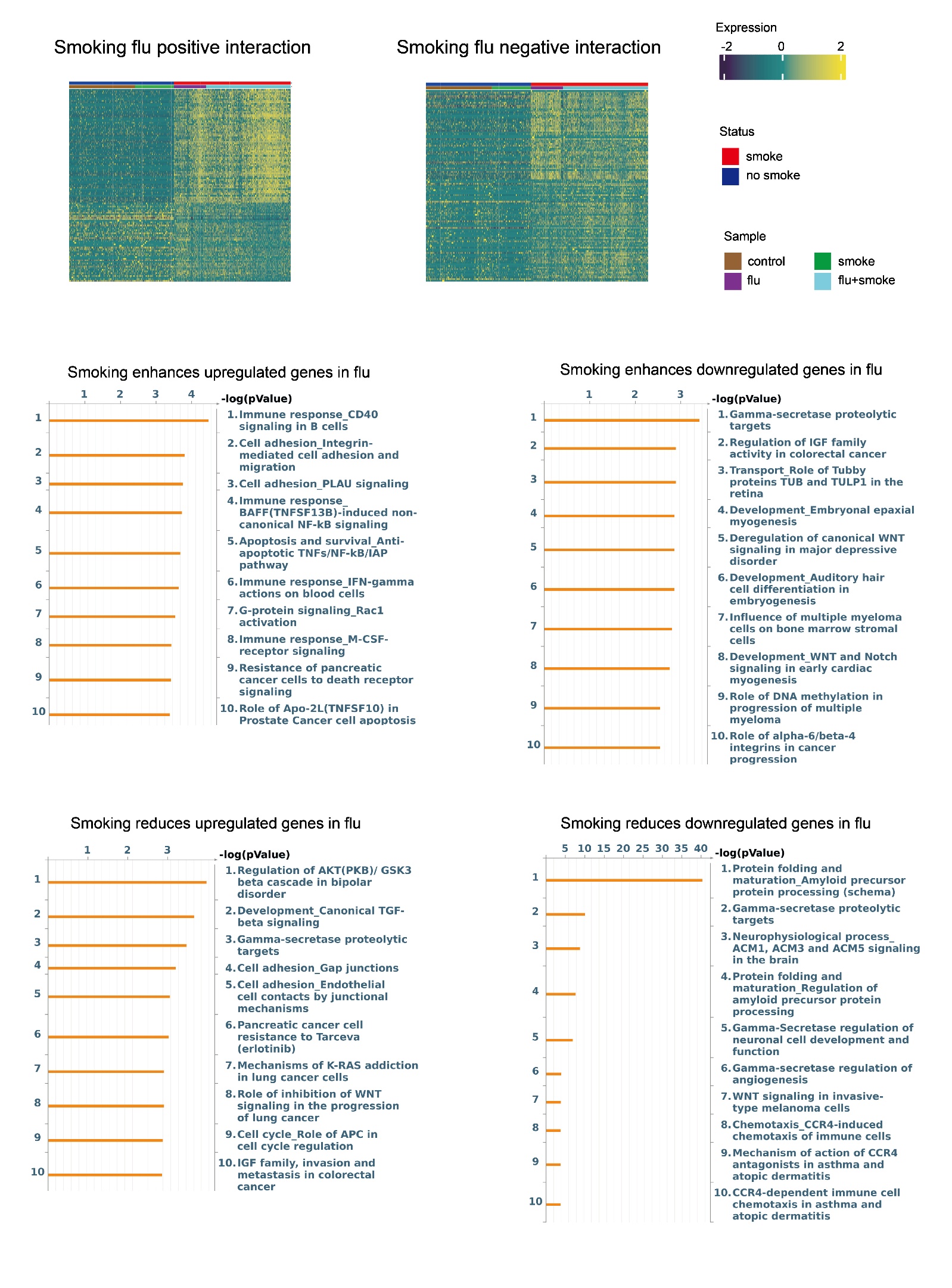


**Supplementary Fig. 2 Direct scRNA–scATAC integration identifies smoking–influenza interactions in ciliated cells.**

Top: Heatmaps show smoking–influenza (flu) interaction genes in ciliated cells. Positively interacting genes (left panels) include cases where smoking enhances the expression of flu-upregulated genes or further suppresses flu-downregulated genes. Negatively interacting genes (right panels) include cases where smoking dampens flu-upregulated genes or counteracts flu-downregulated genes. Significant interaction genes were defined as those differentially expressed in response to flu and further modified by smoking (FDR < 0.05, |log2 fold change| > 0.5). Numbers of interaction genes: smoking enhances flu-up genes (76), enhances flu-down genes (65), reduces flu-up genes (64), reduces flu-down genes (56).

Bottom: Pathway enrichment analysis of positively and negatively interacting smoke–influenza genes. P-values indicate the significance of pathway enrichment

| **Sample** | **Total RNA reads** | **Mean RNA reads per cell** | **Sequencing saturation** | **Valid barcodes** |
| --- | --- | --- | --- | --- |
| **1** | 682,094,113 | 26,896 | 92.50% | 90.40% |
| **2** | 633,144,396 | 35,480 | 95.70% | 88.80% |
| **3** | 502,103,364 | 11,464 | 57.80% | 94.20% |
| **4** | 547,525,062 | 21,461 | 51.20% | 91.90% |
| **5** | 531,751,818 | 48,942 | 88.50% | 91.20% |
| **6** | 546,023,817 | 33,556 | 84.90% | 91.40% |
| **mean** | 573,773,762 | 29,633 | 78.43% | 91.32% |
| **stdev** | 62742481.69 | 11738.82542 | 0.173520092 | 0.01625235 |

**Supplementary Table 1 Summary of single-cell RNA sequencing (scRNA-seq).** Summarizes the scRNA-seq data for six samples, showing an average of 573.8 million total RNA reads, 29,633 mean reads per cell, 78.43% sequencing saturation, and 91.32% valid barcode rates. Sample 1 is control, sample 2 is cigarette smoke (CS), sample 3 is influenza (flu), sample 4 is CS plus flu, sample 5 is hyperoxia and sample 6 is CS plus hyperoxia.

| **Sample** | **AT1** | **AT2** | **Ciliated** | **Secretory** | **Transitional** | **Epi_div** |
| --- | --- | --- | --- | --- | --- | --- |
| **1** | 1564 | 2519 | 1106 | 1296 | 8 | 39 |
| **2** | 714 | 1635 | 653 | 957 | 4 | 11 |
| **3** | 1122 | 3128 | 852 | 723 | 389 | 574 |
| **4** | 443 | 423 | 2354 | 597 | 335 | 377 |
| **5** | 633 | 2352 | 398 | 510 | 43 | 21 |
| **6** | 1017 | 2993 | 653 | 850 | 44 | 9 |

**Supplementary Table 2 Summary of epithelial cell number in each sample.** Sample 1 is control, sample 2 is cigarette smoke (CS), sample 3 is influenza (flu), sample 4 is CS plus flu, sample 5 is hyperoxia and sample 6 is CS plus hyperoxia.

| **Sample** | **Total ATAC reads** | **Mean ATAC reads per cell** | **Sequencing saturation** | **Valid barcodes** |
| --- | --- | --- | --- | --- |
| **1** | 676844825 | 33833.7828 | 69.05% | 97.39% |
| **2** | 715285783 | 42632.3628 | 69.52% | 97.65% |
| **3** | 705565548 | 44937.6185 | 64.41% | 97.4% |
| **4** | 776149682 | 38768.7154 | 65.55% | 97.58% |
| **5** | 488335391 | 52050.2442 | 72.57% | 97.63% |
| **6** | 461509960 | 32169.9401 | 60.36% | 97.38% |
| **mean** | 637281864.8 | 40732.11063 | 66.91% | 97.51% |
| **stdev** | 118797857.5 | 6759.561304 | 0.039671442 | 0.001170114 |

**Supplementary Table 3 Summary of single-cell ATAC sequencing (scATAC-seq) data.** Summarizes the scATAC-seq data for six samples, showing an average of 637.3 million total ATAC reads, 40,732 mean reads per cell, 66.91% sequencing saturation, and 97.51% valid barcode rates. Sample 1 is control, sample 2 is cigarette smoke (CS), sample 3 is influenza (flu), sample 4 is CS plus flu, sample 5 is hyperoxia and sample 6 is CS plus hyperoxia.

| **cell type** | **comparison group** | **regulation** | **DEG number** | **DAP number** | **DEG-DAP pair (direct integration)** | **DEG-DAP pair (TF integration)** |
| --- | --- | --- | --- | --- | --- | --- |
| **AT1** | flu | down | 1,374 | 338 | 81 | 37 |
|  | flu | up | 2,058 | 530 | 120 | 68 |
|  | hyperoxia | down | 1,095 | 347 | 62 | 55 |
|  | hyperoxia | up | 1,747 | 414 | 100 | 50 |
| **AT2** | flu | down | 2,253 | 565 | 164 | 59 |
|  | flu | up | 2,242 | 1,306 | 369 | 217 |
|  | hyperoxia | down | 1,145 | 36 | 11 | 3 |
|  | hyperoxia | up | 2,229 | 414 | 100 | 30 |
| **Ciliated** | flu | down | 1,975 | 2,353 | 480 | 162 |
|  | flu | up | 3,662 | 1,255 | 474 | 225 |
|  | hyperoxia | down | 1,695 | 346 | 66 | 12 |
|  | hyperoxia | up | 2,971 | 2,669 | 571 | 70 |
| **Secretory** | flu | down | 1,240 | 1,299 | 218 | 91 |
|  | flu | up | 2,237 | 485 | 111 | 63 |
|  | hyperoxia | down | 1,513 | 434 | 77 | 9 |
|  | hyperoxia | up | 2,553 | 1,776 | 428 | 76 |

**Supplementary Table 4 Summary of integrating single-cell RNA sequencing (scRNA-seq) and single-cell ATAC sequencing (scATAC-seq).** Summarize the number of differentially expressed genes (DEGs) and differentially accessible peaks (DAPs) and their integration pairs identified through both direct integration and transcription factor (TF) integration across various pulmonary cell types (AT1, AT2, Ciliated, and Secretory) under influenza (flu) and hyperoxia conditions, detailing both upregulated and downregulated regulations for each comparison group.

|  | **AT1** | **AT2** | **Ciliated** | **Secretory** |
| --- | --- | --- | --- | --- |
| **Downregulated genes with smoking** | 61 | 49 | 153 | 61 |
| **Upregulated genes with smoking** | 92 | 69 | 225 | 67 |
| **DAP with decreased accessibility with smoking** | 159 | 155 | 197 | 313 |
| **DAP with increased accessibility with smoking** | 361 | 280 | 1651 | 723 |
| **Downregulated DEG-DAP Pair with smoking**  **(Direct integration / TF integration)** | 0 / 1 | 0 / 0 | 3 / 0 | 5 / 2 |
| **Upregulated DEG-DAP Pair with smoking**  **(Direct integration / TF integration)** | 4 / 3 | 7 / 3 | 25 / 8 | 8 / 6 |

**Supplementary Table 5** Summary of differentially expressed gene (DEG), differentially accessible peak (DAP), differentially expressed gene-differentially accessible peak (DEG-DAP) pair number with smoking.

|  |  | **AT1** | **AT2** | **Ciliated** | **Secretory** |
| --- | --- | --- | --- | --- | --- |
| **DEG** | **Smoking enhances upregulated genes with flu** | 46 | 126 | 226 | 89 |
|  | **Smoking enhances downregulated genes with flu** | 235 | 489 | 297 | 137 |
|  | **Smoking reduces upregulated genes with flu** | 188 | 306 | 461 | 166 |
|  | **Smoking reduces downregulated genes with flu** | 36 | 175 | 247 | 42 |
| **DAP** | **Smoking enhances accessibility with flu** | 3 | 80 | 207 | 5 |
|  | **Smoking further reduces accessibility with flu** | 136 | 379 | 469 | 283 |
|  | **Smoking reduces increased accessibility with flu** | 34 | 107 | 65 | 53 |
|  | **Smoking mitigates decreased accessibility with flu** | 3 | 43 | 299 | 4 |
| **DEG-DAP pair**  **(Direct integration / TF integration)** | **Smoking enhances upregulated genes in flu** | 6 / 4 | 78 / 41 | 76 / 52 | 7 / 13 |
|  | **Smoking enhances downregulated genes in flu** | 20 / 11 | 57 / 19 | 65 / 58 | 12 / 5 |
|  | **Smoking reduces upregulated genes in flu** | 2 / 10 | 87 / 281 | 64 / 229 | 5 / 0 |
|  | **Smoking reduces downregulated genes in flu** | 2 / 1 | 145 / 106 | 56 / 28 | 2 / 0 |

**Supplementary Table 6** Summary of differentially expressed gene (DEG), differentially accessible peak (DAP), differentially expressed gene-differentially accessible peak (DEG-DAP) number in smoking-influenza (flu) interactions.

|  |  | **AT1** | **AT2** | **Ciliated** | **Secretory** |
| --- | --- | --- | --- | --- | --- |
| **DEG** | **Smoking enhances upregulated genes in hyperoxia** | 89 | 100 | 127 | 90 |
|  | **Smoking enhances downregulated genes in hyperoxia** | 52 | 76 | 57 | 91 |
|  | **Smoking reduces upregulated genes in hyperoxia** | 148 | 116 | 212 | 148 |
|  | **Smoking reduces downregulated genes in hyperoxia** | 51 | 44 | 97 | 131 |
| **DAP** | **Smoking enhances accessibility in hyperoxia** | 0 | 0 | 5 | 14 |
|  | **Smoking further reduces accessibility in hyperoxia** | 0 | 4 | 10 | 10 |
|  | **Smoking reduces increased accessibility in hyperoxia** | 310 | 209 | 742 | 328 |
|  | **Smoking mitigates decreased accessibility genes in hyperoxia** | 153 | 22 | 66 | 91 |
| **DEG-DAP pair**  **(Direct integration / TF integration)** | **Smoking enhances upregulated genes in hyperoxia** | 0 / 0 | 0 / 0 | 1 / 0 | 3 / 0 |
|  | **Smoking enhances downregulated genes in hyperoxia** | 0 / 0 | 0 / 0 | 1 / 0 | 2 / 0 |
|  | **Smoking reduces upregulated genes in hyperoxia** | 3 / 0 | 3 / 0 | 17 / 2 | 9 / 2 |
|  | **Smoking reduces downregulated genes in hyperoxia** | 1 / 0 | 1 / 0 | 0 / 0 | 1 / 0 |

**Supplementary Table 7** Summary of differentially expressed gene (DEG), differentially accessible peak (DAP), differentially expressed gene-differentially accessible peak (DEG-DAP) pair number in smoking-hyperoxia interactions.

| **Our data**  **GSE241468** | **TFs associated with smoking-enhanced upregulation of flu DEGs** | **TFs associated with smoking-enhanced downregulation of flu DEGs** | **TFs associated with smoking-reduced upregulation of flu DEGs** | **TFs associated with smoking-reduced downregulation of flu DEGs** |
| --- | --- | --- | --- | --- |
| **Upregulated TFs in smoking** | ETS1, ERG, ERF, ELK1, FEV, ETV3 |  |  | NKX2-3, ETV6, Creb3l2 |
| **Downregulated TFs in smoking** |  | PITX3, NR1I3, Hic1 | Stat2, SOX13, TCF3, PITX3 |  |

**Supplementary Table 8** Transcription factors (TFs) with same directional effect identified in both GSE241468 and TF integration in murine AT2 cells.

| **Our data**  **GSE241468** | **TFs associated with smoking-enhanced upregulation of flu DEGs** | **TFs associated with smoking-enhanced downregulation of flu DEGs** | **TFs associated with smoking-reduced upregulation of flu DEGs** | **TFs associated with smoking-reduced downregulation of flu DEGs** |
| --- | --- | --- | --- | --- |
| **Upregulated TFs in smoking** | RELB (REL), RELA (REL), |  |  | BARHL1, NKX2-5 |
| **Downregulated TFs in smoking** |  | PITX3 | NKX6-3 |  |

**Supplementary Table 9** Transcription factors (TFs) with same directional effect identified in both GSE241468 and TF integration in murine ciliated cells.
