# Supplementary material for: Integrative single-cell RNA and ATAC sequencing reveals the impact of chronic cigarette smoking on lung epithelial responses to influenza and hyperoxia
Source: Respir Res. 2025 Dec 9;27:13. doi: 10.1186/s12931-025-03393-5 (PMC12801856; doi:10.1186/s12931-025-03393-5)
Supplement: Supplementary file 3 — Supplementary Material 3. [file 12931_2025_3393_MOESM3_ESM.docx]

**Description of Additional Supplementary Files**

File Name: Supplementary Data 1-11

Description: This file contains the following 10 data sheets:

Supplementary Data 1: Differentially expressed genes (DEGs) of flu in AT1, AT2, Ciliated and Secretory

Supplementary Data 2: Differentially accessible peaks (DAPs) of flu in AT1, AT2, Ciliated and Secretory

Supplementary Data 3: DEGs of hyperoxia in AT1, AT2, Ciliated and Secretory

Supplementary Data 4: DAPs of hyperoxia in AT1, AT2, Ciliated and Secretory

Supplementary Data 5: DEGs of smoking in AT1, AT2, Ciliated and Secretory

Supplementary Data 6: DAPs of smoking in AT1, AT2, Ciliated and Secretory

Supplementary Data 7: Interacting genes of smoking-flu in AT1, AT2, Ciliated and Secretory

Supplementary Data 8: Interacting peaks of smoking-flu in AT1, AT2, Ciliated and Secretory

Supplementary Data 9: Interacting genes of smoking-hyperoxia in AT1, AT2, Ciliated and Secretory

Supplementary Data 10: Interacting peaks of smoking-hyperoxia in AT1, AT2, Ciliated and Secretory

Supplementary Data 11: Interacting genes of smoking-flu in AT2 (GSE261627); DEGs of smoking in AT2 (GSE241468); DAPs of smoking in AT2 (GSE241468)
